# Hypersensitivity Reactions to Nonsteroidal Anti-Inflammatory Drugs: An Update

**DOI:** 10.3390/ph3010010

**Published:** 2010-01-05

**Authors:** Mario Sánchez-Borges, Fernan Caballero-Fonseca, Arnaldo Capriles-Hulett, Luis González-Aveledo

**Affiliations:** 1 Allergy and Immunology Department, Centro Médico Docente La Trinidad, Caracas, Venezuela; E-Mails: fercab@cantv.net (F.C.-F.); arnaldocapriles@gmail.com; 2 Dermatology Service, Centro Médico de Caracas, Caracas, Venezuela; E-Mail: lagaveledo@gmail.com (L.G.-A.)

**Keywords:** asthma, anaphylaxis, drug reactions, non steroidal anti-inflammatory drugs (NSAIDs), urticaria and angioedema

## Abstract

After beta lactam antibiotics, hypersensitivity reactions to nonsteroidal anti-inflammatory drugs are the second cause of hypersensitivity to drugs. Acute manifestations affect the respiratory tract (aspirin exacerbated respiratory disease), the skin (urticaria and angioedema), or are generalized (anaphylaxis). Correct diagnosis and treatment in order to prevent unnecessary morbidity and the potential risk of death from these severe reactions, and to provide proper medical advice on future drug use frequently requires the participation of allergology specialists familiar with these clinical conditions.

## 1. Introduction

Soon after acetylsalicylic acid was introduced into medical use the first adverse reaction to aspirin was reported by Hirschberg in 1902 [1]. A number of pharmaceutical compounds with anti-inflammatory and analgesic properties, designated nonsteroidal anti-inflammatory drugs (NSAIDs), share the inhibition of cyclooxygenase enzymes (COX) as their main mechanism of action. These enzymes participate in the metabolism of arachidonic acid, resulting in the production of potent inflammatory mediators such as prostaglandins and thromboxanes. 

Two isoenzymes of COX, COX-1 (constitutive form) and COX-2 (inducible form) have been identified. The classical NSAIDs inhibit both isoenzymes and their use is often accompanied by gastrointestinal intolerance due to a decreased production of protective prostaglandin E_2_ in the stomach. 

New drugs that inhibit selectively COX-2 exhibit a better gastric tolerance profile, although their introduction into clinical practice has been associated with severe cardiovascular adverse events that led to the recommendation for careful utilization in patients with previous vascular diseases [2,3]. The preferential COX-2 inhibitors meloxicam and nimesulide are also available in some countries (Table 1). 

**Table 1:** Classification of NSAIDs  according to their  selectivity for COX Isoenzymes.

Selectivity	Drugs
Weak COX inhibitors	Acetaminophen,Salsalate
Inhibitors of COX-1 and COX-2	Acetylsalicylic acid, Piroxicam, Indomethacin, Sulindac, Tolmetin, Ibuprofen, Naproxen, Fenoprofen, Meclofenamate, Mefenamic acid Diflunisal, Ketoprofen, Diclofenac, Ketorolac, Etodolac, Nabumetone, Oxaprozin, Flurbiprofen
Preferential COX-2 inhibitors	Nimesulide, Meloxicam
Selective COX-2 inhibitors	Celecoxib, Rofecoxib,Valdecoxib, Etoricoxib, Parecoxib, Lumiracoxib

This article is an update of the present knowledge on the diverse hypersensitivity reactions that can be induced by NSAIDs, including immediate and delayed reactions. 

## 2. Hypersensitivity Reactions to NSAIDs

The nomenclature for reactions to NSAIDs in the medical literature is somewhat confusing because authors have employed diverse terms in their reports, for example, pseudoallergy, idiosincrasia, or intolerance. We will use the terminology proposed by the Nomenclature Committee of the World Allergy Organization that defines drug hypersensitivity as the symptoms or signs initiated by exposure to a drug at a dose normally tolerated by non-hypersensitive persons. “Drug allergy” refers to immunologically mediated drug hypersensitivity reactions. These may be either immunoglobulin E (IgE)-mediated (immediate) or non IgE-mediated (delayed). “Non allergic hypersensitivity reactions” refer to adverse drug reactions that are not mediated by immunological mechanisms [4].

Analgesic and anti-inflammatory medications are widely used in all age groups for the treatment of pain, inflammation and fevers of diverse etiologies. A large proportion of the population is exposed to these drugs, making them the second cause of untoward reactions, after beta lactam antibiotics.

The prevalence of hypersensitivity to NSAIDs has been estimated to be 0.5 to 1.9% of the general population, whereas NSAIDs are responsible for 21 to 25% of all adverse reactions to drugs. In adult asthmatics aspirin intolerance occurs in 4.3 to 11% of patients and in patients with asthma and nasal polyposis in 25.6%. Aspirin intolerance is present with increased frequency in patients with chronic idiopathic urticaria (CIU) [5,6], and NSAIDs are among the leading causes of anaphylaxis [7]. Additional risk factors for NSAID hypersensitivity are female gender, young adulthood, atopy [8], and intermittent NSAID use for the relief of acute pain [9]. 

## 3. Clinical Picture

Hypersensitivity to NSAIDs can be classified according to the time of onset and the clinical manifestations into acute and delayed. Acute reactions start immediately to several hours after drug administration and include:

(1) Respiratory reactions: Observed in patients with Aspirin Exacerbated Respiratory Disease (AERD), also called aspirin triad, Samter’s disease, or aspirin intolerant asthma. These individuals experience a chronic disease characterized by chronic rhinosinusitis, severe persistent and steroid-dependent asthma, with or without nasal polyposis. Acute asthma exacerbations occur when they receive aspirin or classic NSAIDs. These asthma attacks are severe and may be life-threatening. Various genetic polymorphisms have been associated with this condition [10].
(2) Cross reacting urticaria and angioedema: Exacerbations of urticaria and/or angioedema induced by COX-1 inhibitors are observed in up to one third of patients with chronic urticaria, more often with drugs of the heteroaryl group (naproxen, diclofenac, ibuprofen) [11]. Various genetic polymorphisms, including genes coding for HLA antigens, LTC_4_ synthase, 5-lipooxygenase, and the high affinity receptor for IgE have been observed in these patients [10].
(3) Urticaria, angioedema and anaphylaxis induced by multiple NSAIDs: In patients who do not suffer other morbid conditions NSAIDs can precipitate acute urticaria, angioedema or systemic reactions. This variant of hypersensitivity is more prevalent in atopic individuals [8,12] and facial angioedema is the most frequent clinical manifestation [13]. It has been associated with A444-C allele of LTC_4 _synthase [14].
(4) Urticaria, angioedema and anaphylaxis induced by a single NSAID: More frequently triggered by pyrazolones, but also reported for aspirin, paracetamol, ibuprofen, diclofenac and naproxen. These reactions constitute about 30% of adverse reactions to NSAIDs and are observed with increased frequency in patients with previous history of atopic disease, food or drug allergy. The clinical manifestations include urticaria, angioedema, laryngeal edema, anaphylaxis, generalized pruritus, rhinitis or bronchospasm.

Delayed reactions begin after 24 hours of NSAID exposure, can be induced by a single or multiple cross-reacting NSAIDs, and are clinically expressed either as organ specific or as multisystemic diseases [15]. Examples of organ specific diseases are:

(1) Skin: Maculopapular exanthemas, Fixed drug eruptions [16], Bullous reactions (erythema multiforme, Stevens-Johnson syndrome, toxic epidermal necrolysis) [17], acute generalized exanthematous pustulosis [18], contact and photocontact dermatitis [19,20].
(2) Lung: Pneumonitis [21].
(3) Central nervous system: Aseptic meningitis [22].
(4) Kidney: Nephritis [23].

## 4. Pathogenesis

Since NSAID hypersensitivity has multiple clinical manifestations, the mechanisms incriminated in each of them are different. Reactions to aspirin and NSAIDs observed in patients with AERD are mediated by inhibition of COX-1, leading to a shunting of arachidonic acid metabolism towards the 5-lipoxygenase pathway and increased production of cysteinyl leukotrienes [24]. A decreased production of PGE_2_, a modulator of mediator release from mast cells and other inflammatory cells, also plays a role [25]. 

Urticaria and angioedema exacerbations in patients with CIU are also mediated by COX-1 inhibition, as suggested by Mastalerz *et al*. They observed increased levels of urinary LTE_4 _(uLTE_4_) in patients with chronic urticaria that showed symptom exacerbations during challenges with aspirin, as compared with CIU patients that did not respond to the aspirin provocation. Further increases of uLTE_4_ occurred in the first group, but not in tolerant patients, after ASA challenge [26].

The mechanisms of induction of acute urticaria, angioedema and anaphylaxis by multiple NSAIDs in patients who do not have chronic urticaria have not been defined. Nevertheless, it is provocative to hypothesize that, since these drugs have as their major mean of action the inhibition of COX-1, this mechanism could also be involved in the production of these clinical manifestations.

Reactions to a single NSAID are mediated by drug-specific IgE antibodies, as can be demonstrated by means of immediate-type skin tests or *in vitro* measurement of specific IgE [27,28]. 

Delayed reactions to NSAIDs are mediated by drug-specific cytotoxic T cells through type IV allergic reactions. These can be subclassified in 4 subtypes (IVa, IVb, IVc and IVd) according to the main effector cells involved in their production (monocytes, eosinophils, CD4 and CD8 lymphocytes, or neutrophils [29].

## 5. Diagnosis

The information on symptoms and exposure to NSAIDs is of paramount importance to determine the temporal relationship between the initiation of the clinical picture and the probability of a drug etiology. In general, in patients who show repeated episodes of urticaria, angioedema or asthma after receiving one or various cross-reacting NSAIDs, the medical history is reliable. Confirmation is generally obtained by oral provocation tests, single- or double-blinded, according to the protocols shown in Table 2. 

**Table 2:** Oral provocation tests with acetylsalicylic acid (ASA).

Patients with respiratory symptoms (AERD)			
Hour	Day 1	Day 2	Day 3
08:00	Placebo	ASA 3 or 30 mg	ASA 150 mg
11:00	Placebo	ASA 60 mg	ASA 325 mg
14:00	Placebo	ASA 100 mg	ASA 650 mg
Monitor pulmonary function, naso-ocular symptoms/signs			
Test positive if a decrease of FEV1 ° 20 % is observed			
**Patients with urticaria and/or angioedema**			
08:00	Placebo	ASA 100 mg	ASA 325 mg
10:00	Placebo	ASA 200 mg	ASA 650 mg
Skin scores recorded every 2 hours			

These tests are better performed in the hospital by trained specialists, with easily available drugs and equipment to treat reactions if they occur. In Europe, inhalation or nasal challenges with lysine-aspirin are also being used routinely for the diagnosis of AERD [30]. 

Recently De Weck and coworkers proposed an *in vitro* test suitable for the study of NSAID hypersensitivity. The basophil activation test (BAT) is based on the flow cytometric quantification of the mast cell activation marker CD63 when patient’s leukocytes are incubated with the drug *in vitro*. The sensitivity of this test is 75% and the specificity ranges between 45 and 95 %. This is a promising method for those patients in whom a drug provocation is not possible due to ethical or clinical reasons [31,32], but is not available in most centers since it requires alive blood cells (less than 24 hours of sample drawing) and a fluorescence-activated cell sorter (FACS).

An alternative test under study is based on the aspirin-induced release of LTC4 from peripheral blood leukocytes, although its sensitivity is lower than BAT’s [33]. A third *in* *vitro* test under development is the ASPI TEST® that measures 15-hydroxyeicosatetranoic acid (15-HETE) release induced by ASA from peripheral blood leukocytes. Its sensitivity in patients with AERD is 82% and its specificity 83% [34]. 

Prick and intradermal skin tests with pyrazolone, paracetamol and diclofenac have been used in patients with single-drug hypersensitivity, but at this time these tests have not been standardized for general use [35,36,37].

Patch and photopatch tests are a simple and fast method for the diagnosis of delayed reactions to NSAIDs [38,39], and concentrations of NSAIDs for patch testing have been previously published [40]. Intradermal and scratch tests with reading at 48 hours are also useful [41]. The lymphocyte transformation test measures the in vitro proliferative response of T cells to the drug. The test is available in few centers, is costly and laborious. Rechallenges with the drug are considered the gold standard for the diagnosis of delayed reactions to NSAIDs although they are contraindicated in patients with previous severe reactions.

## 6. Patient Management

Patients with AERD must avoid all COX-1 inhibitors, including aspirin, in order to avoid the occurrence of serious asthma exacerbations. For the treatment of pain and inflammation NSAIDs that do not inhibit COX-1, such as acetaminophen in doses below 1,000 mg and COX-2 inhibitors are recommended after challenge in the office or medical facility. Aspirin desensitization is indicated for patients who require continuous anti-inflammatory or anti-thrombotic therapy, such as those with ischemic heart disease or chronic arthritis [42]. Persistent asthma and rhinosinusitis are to be treated according to the recommendations given by international guidelines such as GINA and EPOS [43,44].

Patients with CIU intolerant to ASA/NSAIDs should avoid all inhibitors of COX-1. Alternative drugs such as acetaminophen (tolerated by about 89% of these patients), or COX-2 inhibitors may be used after single-blinded oral challenge [45,46]. The treatment of chronic urticaria has been recently updated and is based on the use of non sedating antihistamines alone or in combination with other drugs [47].

Patients with urticaria, angioedema and anaphylaxis precipitated by multiple NSAIDs are advised to avoid COX-1 inhibitors. Acetaminophen and COX-2 inhibitors are alternative drugs suitable for analgesia and treatment of pain and inflammation in these subjects. COX-2 inhibitors are not recommended for chronic use because of the increased risk of cardiovascular side effects, especially in patients with previous history of coronary or cerebrovascular disease.

For patients with reactions to a single drug, avoidance of the drug and other NSAIDs chemically related should be recommended. These patients can be treated with other non cross-reacting NSAIDs (Table 3).

**Table 3:** Chemical Classification of non steroidal Anti-inflammatory Drugs (NSAIDs).

Group	Drugs
Salicylic acid derivatives	Aspirin, sodium salicylate, choline magnesium trysalicylate, salsalate, diflunisal, salicilsalicylic acid, sulfasalazine, olsalazine
Para-aminophenol derivatives	Acetaminophen
Indol and indene acetic acids	Indomethacin, sulindac, etodolac
Heteroaryl acetic acid	Tolmetin, diclofenac, ketorolac
Arilpropionic acid	Ibuprofen, naproxen, flurbiprofen, ketoprofen, fenoprofen, oxaprozin
Antranilic acid (fenamates)	Mefenamic acid, meclofenamic acid
Enolic acid	Oxicams (piroxicam, tenoxicam), pyrazoledinediones (fenilbutazone, oxyfentathrazone)
Alkanones	Nabumetone
Pyrazolic derivatives	Antipyrin, aminopyrin, dipyrone

## 7. Conclusions

NSAIDs constitute a frequent cause of adverse reactions to drugs that can be clinically manifested in multiple forms. Acute reactions may be systemic (anaphylaxis), respiratory (aspirin- exacerbated respiratory disease), and cutaneous (urticaria and angioedema). Delayed reactions include various types of skin conditions, or the involvement of various organs such as the lungs, central nervous system or the kidneys. Diagnosis of NSAID hypersensitivity generally requires the performance of confirmatory tests in patients with a suggestive clinical picture. Patient management includes drug avoidance, the use of alternative NSAIDs for the relief of pain, fever and inflammation, the treatment of associated diseases such as asthma, rhinosinusitis and urticaria/angioedema. In selected patients with AERD desensitization is indicated.
